# Clinical significance of altered expression of mitochondrial genome-derived LncRNA LIPCAR in colorectal cancer

**DOI:** 10.1016/j.bbrep.2025.102179

**Published:** 2025-07-25

**Authors:** Seyed Taha Nourbakhsh, Fatemeh Mohamadhashem, Mohammad Mehdi Naghizadeh, Amirnader Emami Razavi, Abdolreza Daraei, Faezeh Mohamadhashem

**Affiliations:** a Student Research Committee, Shahrekord University of Medical Sciences, Shahrekord, Iran; b Department of Internal Medicine, Sina Hospital, Tehran University of Medical Sciences, Tehran, Iran; c Noncommunicable Diseases Research Center, Fasa University of Medical Sciences, Fasa, Iran; d Iran National Tumor Bank, Cancer Institute of Iran, Tehran University of Medical Sciences, Tehran, Iran; e Cellular and Molecular Biology Research Center, Health Research Institute, Babol University of Medical Sciences, Babol, Iran; f Department of Medical Genetics, School of Medicine, Babol University of Medical Sciences, Babol, Iran; g Cellular and Molecular Research Center, Basic Health Sciences Institute, Shahrekord University of Medical Sciences, Shahrekord, Iran

**Keywords:** LIPCAR, Mt-lncRNA, Colorectal cancer, Metabolic reprogramming

## Abstract

**Background:**

The prevalence and mortality of colorectal cancer (CRC) are rising; therefore, understanding its molecular pathophysiology is necessary for identifying reliable diagnostic and therapeutic markers. Several studies corroborate the fact that the initiation and progression of various diseases, including cancers, are significantly influenced by the dysregulation of mitochondrial transcripts, such as lncRNAs encoded by the mitochondrial genome. This study is the first to examine the expression profile of LIPCAR in CRC and its correlation with clinicopathological parameters.

**Methods:**

In this work, 40 pairs of CRC tissues were obtained, including 40 tumor samples and 40 adjacent non-tumor samples. The SYBR green technique was applied in real-time PCR to analyze the expression profile of LIPCAR in CRC patients.

**Results:**

The findings indicated a significant downregulation of LIPCAR in tumor tissue samples compared to adjacent non-tumoral tissues (p-value<.05). Furthermore, no significant relationship between the patients' clinical data and decreased LIPCAR expression was found. Receiver Operating Characteristic (ROC) curve analysis revealed that LIPCAR had an area under the curve (AUC) of .75 (p-value <.0001), with a sensitivity of 87.5 % and a specificity of 57.5 % at a cutoff value of .094.

**Conclusion:**

The marked downregulation of LIPCAR expression suggests its critical involvement in the pathogenesis of CRC, potentially functioning as a tumor suppressor. Furthermore, LIPCAR may serve as a clinical biomarker for CRC patients. However, this hypothesis necessitates further validation through comprehensive functional studies conducted both in vitro and in vivo.

## Highlights

• LIPCAR expression was down-regulated in colorectal cancer (CRC).
• No significant association was observed between down regulation of LIPCAR and clinical data.
• LIPCAR has good diagnostic biomarker potential in CRC (AUC = .75).

## 1 Introduction

Colorectal cancer (CRC) ranks as the second-leading cause of cancer-related mortality and the third-most prevalent malignancy worldwide [1]. By 2030, global projections estimate over 2.2 million new CRC cases and 1.1 million deaths [2]. Similar to other solid tumors, CRC is a heterogeneous disease characterized by various subtypes, each defined by unique molecular and/or clinical features. These subtypes fall into three major categories: chromosomal instability (CIN), microsatellite instability (MSI), and CpG island methylation phenotype (CIMP), based on distinct carcinogenetic pathways [3]. Furthermore, CRC can be classified into two primary types: the more common sporadic form, which accounts for approximately 65 % of cases and arises from genetic and epigenetic alterations, and the rarer hereditary forms [4,5]. Despite advances in therapeutic approaches, CRC remains associated with poor prognosis. Consequently, a deeper understanding of the mechanisms driving CRC carcinogenesis and metastasis is essential to identify novel therapeutic targets and biomarkers that enable efficient early detection, personalized treatments, and effective monitoring to improve patient outcomes.

Metabolic reprogramming, a hallmark of cancer, plays a pivotal role in the initiation and progression of various malignancies, including CRC. Cancer cells often rewire their metabolic pathways to sustain rapid proliferation, meeting their substantial energy and biosynthetic demands [6]. One prominent feature of metabolic reprogramming is the Warburg effect, wherein cancer cells preferentially utilize glycolysis over mitochondrial oxidative phosphorylation for energy production, even under oxygen-rich conditions. This metabolic shift is closely tied to mitochondrial dynamics [[7], [8], [9], [10]]. As the metabolic hub of tumor cells, mitochondria fulfill the energy, metabolic, and signaling demands necessary for tumor growth, survival, and metastasis. Thus, mitochondria are integral players in metabolic reprogramming and cancer development [11]. Numerous studies have demonstrated that mitochondrial DNA (mtDNA) mutations and alterations significantly contribute to CRC pathophysiology [[12], [13], [14]].

Recent research has further highlighted the role of mitochondrial transcriptomics in cancer. Beyond mutations in mtDNA, alterations in mitochondrial-encoded non-coding RNAs (mt-ncRNAs) have emerged as significant contributors to the pathogenesis of various diseases, including cancer [[15], [16], [17], [18], [19]]. Notably, a significant subtype of these non-coding RNAs is mitochondrial-encoded long non-coding RNAs (mt-lncRNAs), which, similar to long non-coding RNAs encoded by the nuclear genome, exceed 200 nucleotides in length [20,21].

Long intergenic noncoding RNA predicting cardiac remodeling (LIPCAR), a 781-nucleotide chimeric mt-lncRNA, is one of the most important lncRNAs encoded by the mitochondrial genome. Its 5′ segment corresponds to the antisense strand of the mitochondrial *CYTB* gene, while its 3′ segment aligns with the antisense strand of the mitochondrial *COX2* gene [22]. LIPCAR has been shown to serve as a prognostic biomarker in heart failure and cardiac remodeling [22,23]. Interestingly, elevated LIPCAR expression levels have been reported in hepatocellular carcinoma (HCC), where it promotes tumor growth, migration, and metastasis, suggesting that its biological functions are closely linked to mitochondrial dynamics [24].

To our knowledge, no research has explored LIPCAR expression and its function in CRC. In this study, we aimed to evaluate the clinical relevance of LIPCAR by analyzing its expression profile in CRC tumor tissues versus adjacent non-tumoral tissues and assessing its association with clinicopathological features.

## 2 Materials and methods

### 2.1 Subjects

Forty paired tissue samples (tumoral and adjacent non-tumoral tissues) from patients diagnosed with CRC were obtained from Iran National Tumor Bank (Imam Khomeini Hospital, Tehran, Iran). All patients provided written informed consent before participating and the study was approved by the Ethics Committee of Shahrekord University of Medical Sciences (IR.SKUMS.MED.REC.1402.075). Before surgery, none of the patients had received any preoperative therapies, such as chemotherapy or radiation.

### 2.2 RNA extraction and quantification

Total RNA was extracted from the CRC and adjacent non-tumoral tissues using the RNSol H reagent (ROJE Technologies, Tehran, Iran), according to the manufacturer's protocol. The quality and quantity of the RNA were assessed using both spectrophotometric analysis (NanoDrop 2000c, Thermo Fisher Scientific) and electrophoresis on a 1 % agarose gel. cDNA synthesis was carried out using Easy cDNA Synthesis Kit (Parstous, Mashhad, Iran), following the provided guidelines.

### 2.3 Quantitative real-time PCR

Gene expression levels were quantified using quantitative real-time PCR (qRT-PCR) with specific primers for LIPCAR and *ACTB* as the housekeeping gene. The primers were selected based on previously published studies [25,26]. Reactions were carried out in duplicate using Rotor-Gene 6000 (Qiagen, Hilden, Germany) and ExcelTaq 2X Q-PCR Master Mix (SMOBIO). The amplification program included an initial denaturation at 95 °C for 10 min, followed by 40 cycles of 95 °C for 15 s, 59 °C for 30 s (annealing temperature), and 72 °C for 30 s. The relative expression of LIPCAR was calculated using the 2^−ΔΔCt^ method.

### 2.4 Statistical analysis

IBM SPSS Statistics 27 was used for statistical analysis, and *p*-values <.05 were considered statistically significant. The relative expression of LIPCAR in tumors and nearby non-tumoral tissues was compared using the Wilcoxon test and the paired sample *t*-test. The relationship between the LIPCAR fold change and clinicopathological data was investigated using the Mann-Whitney and Kruskal-Wallis tests. Based on the median, the LIPCAR fold change was then divided into two groups: low and high expression. The difference between these groups was examined using the chi-square test. The Receiver Operating Characteristic (ROC) curve and the area under the curve (AUC) were used to assess the diagnostic value, while the Kaplan–Meier method was employed to evaluate overall survival (OS). Graphs were created using Excel software.

## 3 Results

### 3.1 Clinical characteristics

This study included 40 CRC patients. The clinical and demographic characteristics of the study population are summarized in Table 1. The study comprised 70 % males and 30 % females, with a mean age of 59.33 years (range: 26–93 years). Most patients presented with tumors classified as T3-T4 (87.5 %), N1–N2 (80 %) with no evidence of metastasis (M0:95 %). Histopathological evaluation revealed that 80 % of cases were categorized as stages III-IV, and lymph node involvement was observed in 65 %. Additionally, lifestyle factors such as smoking were reported in 20 % of patients. No significant differences were observed between the groups in any of these variables.

**Table 1:** Demographic and clinicopathological parameters of CRC participants.

		Count	Column N %
Age	<60	21	52.5 %
	>60	19	47.5 %
Sex	F	12	30.0 %
	M	28	70.0 %
Site of primary	Colon	26	65.0 %
	Rectum	14	35.0 %
Grade	Grade I	10	25.0 %
	Grade II	20	50.0 %
	Grade III	10	25.0 %
Lymphatic invasion	Yes	26	65.0 %
	No	14	35.0 %
Vascular invasion	Yes	28	70.0 %
	No	12	30.0 %
Pathological T	T1-T2	5	12.5 %
	T3-T4	35	87.5 %
Pathological N	N0	8	20.0 %
	N1–N2	32	80.0 %
Clinical Metastasis	M0	38	95.0 %
	M1	2	5.0 %
Stage	I-II	8	20.0 %
	III-IV	32	80.0 %
Smoking Status	Non-smoker	32	80.0 %
	Smoker	8	20.0 %

**Table 2:** Association between the clinicopathological and demographic features of CRC patients and LIPCAR expression.

		LIPCAR				P-value
		Number	Mean	SD	Median	
Age	<60	21	.48	.50	.28	.49
	>60	19	.66	.81	.32	
Sex	F	12	.60	.50	.58	.55
	M	28	.56	.73	.28	
Site of primary	Colon	26	.41	.41	.23	.06
	Rectum	14	.87	.92	.57	
Histology grade	Grade I	10	.49	.42	.45	.79
	Grade II	20	.54	.75	.25	
	Grade III	10	.71	.72	.41	
Lymphatic invasion	Yes	26	.52	.70	.26	.44
	No	14	.67	.60	.65	
Vascular invasion	Yes	28	.51	.68	.26	.4
	No	12	.70	.62	.65	
Pathological T	T1-T2	5	.94	.51	.95	.05
	T3-T4	35	.52	.67	.28	
Pathological N	N0	8	.72	.62	.50	.23
	N1–N2	32	.53	.68	.26	
Clinical Metastasis	M0	38	.56	.68	.28	.19
	M1	2	.79	.34	.79	
Stage	I-II	8	.72	.62	.50	.23
	III-IV	32	.53	.68	.26	
Smoking Status	Non-smoker	32	.49	.47	.26	.41
	Smoker	8	.90	1.14	.48

**Table 3:** Association between the clinicopathological and demographic features of CRC patients and LIPCAR expression, according to dividing fold changes into two groups of high and low expressions.

		LIPCAR				P-value
		Low		High		
		Count	Column N %	Count	Column N %	
Age	<60	12	60.0 %	9	45.0 %	.34
	>60	8	40.0 %	11	55.0 %	
Sex	F	5	25.0 %	7	35.0 %	.49
	M	15	75.0 %	13	65.0 %	
Site of primary	Colon	15	75.0 %	11	55.0 %	.18
	Rectum	5	25.0 %	9	45.0 %	
Histology grade	Grade I	4	20.0 %	6	30.0 %	.44
	Grade II	12	60.0 %	8	40.0 %	
	Grade III	4	20.0 %	6	30.0 %	
Lymphatic invasion	Yes	14	70.0 %	12	60.0 %	.5
	No	6	30.0 %	8	40.0 %	
Vascular invasion	Yes	15	75.0 %	13	65.0 %	.49
	No	5	25.0 %	7	35.0 %	
Pathological T	T1-T2	1	5.0 %	4	20.0 %	.15
	T3-T4	19	95.0 %	16	80.0 %	
Pathological N	N0	2	10.0 %	6	30.0 %	.11
	N1–N2	18	90.0 %	14	70.0 %	
Clinical Metastasis	M0	20	100.0 %	18	90.0 %	.14
	M1	0	.0 %	2	10.0 %	
Stage	I-II	2	10.0 %	6	30.0 %	.11
	III-IV	18	90.0 %	14	70.0 %	
Smoking Status	Non-smoker	17	85.0 %	15	75.0 %	.42
	Smoker	3	15.0 %	5	25.0 %	

### 3.2 The expression levels of LIPCAR in tumoral and non-tumoral adjacent tissues

**Fig. 1:**
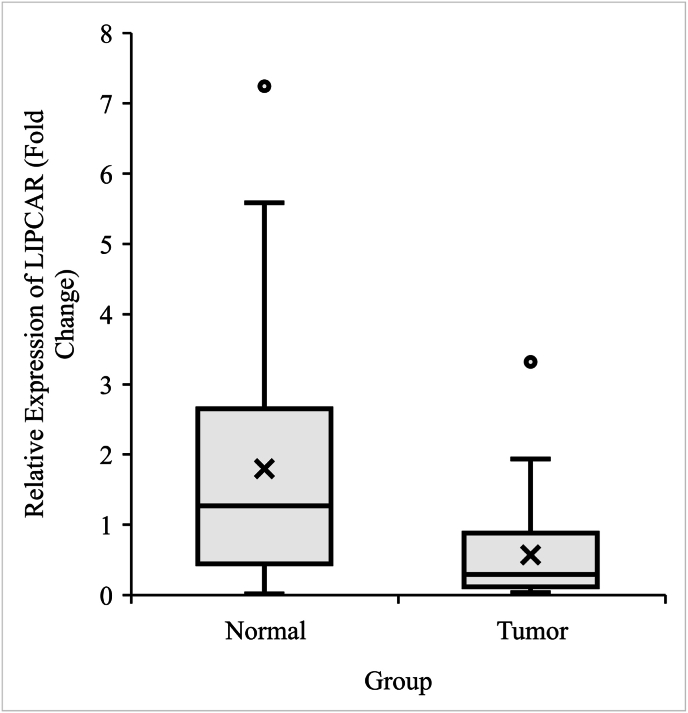
Relative expression level of LIPCAR in CRC samples (n = 40) compared to adjacent non-tumoral samples (n = 40). The paired sample *t*-test was used to compare the level of LIPCAR expression. SCAR/mc-COX2 expression was significantly decreased (*p*-value = 0001) in tumor tissues compared to adjacent non-tumoral tissues.

The statistical analysis of the qRT-PCR data revealed a significant downregulation of LIPCAR in tumor tissues (median = .29) compared to the adjacent non-tumoral tissues (median = 1.26; *p*-value = .0001; Fig. 1).

### 3.3 Clinical significance and diagnostic value of LIPCAR

**Fig. 2:**
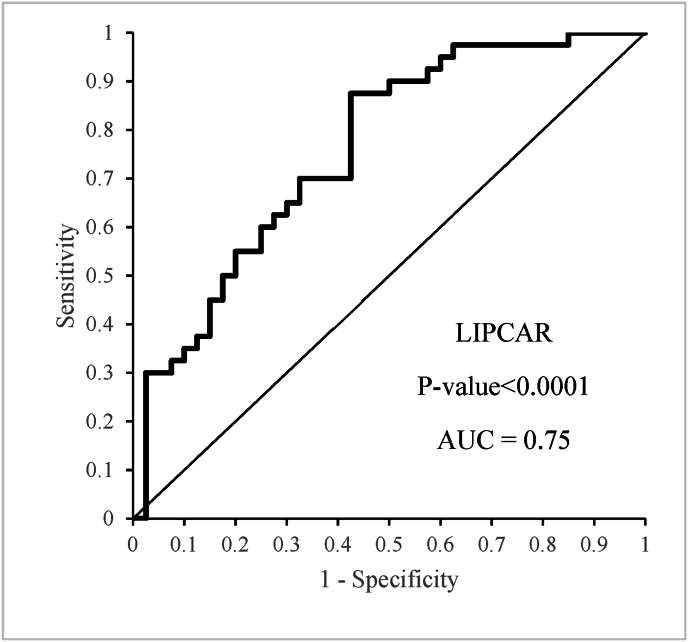
ROC curve analysis on the relative expression level of LIPCAR to ascertain its biomarker value to distinguish tumors from non-tumoral samples.

The potential of LIPCAR as a clinical biomarker was assessed through ROC curve analysis. The findings demonstrated that LIPCAR could be utilized for CRC diagnosis and prognosis, showing an AUC of .75 (p-value <.0001; Fig. 2), with a sensitivity of 87.5 % and a specificity of 57.5 % at a cutoff value of .094. The analysis of the relationship between LIPCAR expression levels and clinicopathological parameters revealed no statistically significant associations (Table 2). Furthermore, categorizing patients into low and high expression groups based on LIPCAR fold change did not demonstrate any significant correlations with clinical characteristics (Table 3).

## 4 Discussion

Metabolic pathways are critical for maintaining cellular stability and homeostasis under normal physiological conditions. However, during the onset and progression of cancer, these pathways undergo significant alterations that become crucial for the survival and proliferation of cancer cells, as well as for the process of carcinogenesis [27]. Consequently, metabolic reprogramming has emerged as a hallmark of many malignancies, including CRC [6,28]. Mitochondria, as one of the most vital intracellular organelles, play diverse roles in cellular processes, including energy production, metabolite synthesis, regulation of cell signaling and apoptosis, and maintenance of metabolic homeostasis [[29], [30], [31]]. Extensive research has identified mitochondrial dysfunction as a fundamental factor in CRC development. Mitochondrial alterations drive carcinogenesis by reshaping metabolic pathways, facilitating tumor initiation and growth, promoting resistance to therapeutic agents, and enhancing metastatic potential [10,32]. Advances in molecular techniques and bioinformatics tools have further underscored the role of mitochondrial transcriptomics in cancer. Beyond the well-documented contributions of mtDNA defects and mutations, disruptions in mitochondrial transcriptomics—including non-coding RNAs, such as mt-lncRNAs—have been increasingly recognized as pivotal players in cancer pathogenesis [17,[33], [34], [35], [36], [37]].

In this study, we investigated the expression level of the important mt-lncRNA LIPCAR in CRC tumor tissues to adjacent non-tumoral tissues. Additionally, we explored its potential relationship with clinicopathological factors. Our results revealed a significant downregulation of LIPCAR expression in tumor tissues. However, no significant correlation was observed between LIPCAR expression and clinicopathological parameters. Furthermore, Receiver Operating Characteristic (ROC) curve analysis demonstrated that LIPCAR exhibited promising biomarker potential, with an Area Under the Curve (AUC) value of .75. Notably, the addition of other clinicopathological characteristics to the expression variable, followed by ROC curve analysis, improves the accuracy of biomarker discovery; however, the sample size in this study precluded this.

Given its reduced expression in CRC, LIPCAR may play a significant role in the development and progression of this malignancy, potentially functioning as a tumor suppressor. Interestingly, in a study by Bongolo et al., LIPCAR was shown to be significantly upregulated in hepatocellular carcinoma (HCC) cell lines and plasma samples [24]. Their findings revealed a significant upregulation of LIPCAR expression in both HCC cell lines and plasma samples. Moreover, this elevated expression was associated with various clinicopathological factors, including gender, glucose levels, alanine aminotransferase (ALT), and aspartate aminotransferase (AST). The heightened expression levels demonstrated considerable diagnostic potential for HCC. Notably, the overexpression of LIPCAR significantly promoted cell proliferation, migration, invasion, and epithelial-mesenchymal transition (EMT) while suppressing apoptosis in vitro. Furthermore, it enhanced tumor proliferation and metastatic potential in vivo [24]. Beyond cancer, LIPCAR has been implicated in other pathological conditions. For instance, Zhang et al. reported that elevated plasma levels of LIPCAR were associated with an increased risk of coronary artery disease (CAD), suggesting its potential as a biomarker for this condition [38]. Additionally, LIPCAR levels were shown to rise during advanced stages of left ventricular remodeling and in patients with chronic heart failure, making it a valuable prognostic marker for cardiac remodeling and a predictor of mortality in heart failure patients [22,23]. According to a recent research report, the expression level of LIPCAR in atrial fibrillation was strongly linked to the phosphorylation of TGF-β1 and Smad2/3, indicating that it controls atrial fibrosis through the TGF-β/Smad pathway [39]. Furthermore, Wang et al. demonstrated that vascular smooth muscle cell migration and proliferation were enhanced by LIPCAR overexpression [40]. Notably, one of the main limitations of the present study is the relatively small sample size and the uneven distribution of samples across key clinical parameters, such as tumor stage and grade. Moreover, this study focused solely on comparing LIPCAR expression levels between tumor tissues and their adjacent normal counterparts. To address these limitations, future research should incorporate larger and more clinically diverse patient cohorts, accompanied by detailed clinicopathological data. Such studies would enable a more comprehensive assessment of potential associations between LIPCAR expression and clinical features, thereby providing deeper insights into the biomarker's role in tumor progression and its prognostic significance.

## 5 Conclusion

In conclusion, this study is the first to evaluate the expression levels and clinical significance of LIPCAR in CRC. Our findings indicate that the expression of this mt-lncRNA is significantly downregulated in tumor tissues compared to adjacent non-tumoral tissues. Although no significant associations were observed between LIPCAR expression and clinicopathological characteristics, our results suggest that LIPCAR may hold potential as a biomarker for CRC. However, given the limitations of this study, further functional research is necessary to elucidate the biological role and clinical utility of LIPCAR in CRC.

## Ethics approval and consent to participate

The experimental procedures were approved by the Ethics Committee of Shahrekord University of Medical Sciences (IR.SKUMS.MED.REC.1402.075). Informed consent was obtained from all patients by the Iran National Tumor Bank.

## Consent for publication

Not Applicable.

## Availability of data and materials

Data supporting the findings of this study are available from the corresponding author upon reasonable request.

## Author contributions

Faezeh Mohamadhashem Supervision, Project administration, Funding acquisition, Writing - Review & Editing A Daraei Conceptualization, Writing - Review & Editing ST Nourbakhsh Investigation, Writing - Original Draft Fatemeh Mohamadhashem Writing - Review & Editing M Naghizadeh Formal analysis AN Emami Razavi Resources.

## Fundings

The authors would like to acknowledge the 10.13039/501100005756Shahrekord University of Medical Sciences (SKUMS-6982) for their financial support. The funders had no role in the study design, data collection, analysis, publication decision, or manuscript writing process. The authors have no other relevant affiliations or financial involvement with any organization or entity with a financial interest in or conflict with the subject matter or materials discussed in the manuscript apart from those disclosed.

## Declaration of competing interest

The authors declare that they have no known competing financial interests or personal relationships that could have appeared to influence the work reported in this paper.

## Data Availability

Data will be made available on request.

## References

[1] Sung H., Ferlay J., Siegel R.L., Laversanne M., Soerjomataram I., Jemal A. (2021). Global cancer statistics 2020: GLOBOCAN estimates of incidence and mortality worldwide for 36 cancers in 185 countries. CA Cancer J. Clin..

[2] Arnold M., Sierra M.S., Laversanne M., Soerjomataram I., Jemal A., Bray F. (2017). Global patterns and trends in colorectal cancer incidence and mortality. Gut.

[3] Ogino S., Goel A. (2008). Molecular classification and correlates in colorectal cancer. J. Mol. Diagn..

[4] Jasperson K.W., Tuohy T.M., Neklason D.W., Burt R.W. (2010). Hereditary and familial Colon cancer. Gastroenterology.

[5] Keum N., Giovannucci E. (2019). Global burden of colorectal cancer: emerging trends, risk factors and prevention strategies. Nat. Rev. Gastroenterol. Hepatol..

[6] Hanahan D., Weinberg R.A. (2011). Hallmarks of cancer: the next generation. Cell.

[7] Sessions D.T., Kashatus D.F. (2021). Mitochondrial dynamics in cancer stem cells. Cell. Mol. Life Sci..

[8] Bonnay F., Veloso A., Steinmann V., Kocher T., Abdusselamoglu M.D., Bajaj S. (2020). Oxidative metabolism drives immortalization of neural stem cells during tumorigenesis. Cell.

[9] Li T., Han J., Jia L., Hu X., Chen L., Wang Y. (2019). PKM2 coordinates glycolysis with mitochondrial fusion and oxidative phosphorylation. Protein Cell.

[10] Wu Z., Xiao C., Long J., Huang W., You F., Li X. (2024). Mitochondrial dynamics and colorectal cancer biology: mechanisms and potential targets. Cell Commun. Signal..

[11] Vasan K., Werner M., Chandel N.S. (2020). Mitochondrial metabolism as a target for cancer therapy. Cell Metab..

[12] Sun X., Zhan L., Chen Y., Wang G., He L., Wang Q. (2018). Increased mtDNA copy number promotes cancer progression by enhancing mitochondrial oxidative phosphorylation in microsatellite-stable colorectal cancer. Signal Transduct. Targeted Ther..

[13] Guo W., Liu Y., Ji X., Guo S., Xie F., Chen Y. (2023). Mutational signature of mtDNA confers mechanistic insight into oxidative metabolism remodeling in colorectal cancer. Theranostics.

[14] Wu Z., Zuo M., Zeng L., Cui K., Liu B., Yan C. (2021). OMA1 reprograms metabolism under hypoxia to promote colorectal cancer development. EMBO Rep..

[15] Jusic A., Devaux Y., Action E.U.-C.C. (2020). Mitochondrial noncoding RNA-regulatory network in cardiovascular disease. Basic Res. Cardiol..

[16] Wu Z., Sun H., Wang C., Liu W., Liu M., Zhu Y. (2020). Mitochondrial genome-derived circRNA mc-COX2 functions as an oncogene in chronic lymphocytic leukemia. Mol. Ther. Nucleic Acids.

[17] Liu X., Wang X., Li J., Hu S., Deng Y., Yin H. (2020). Identification of mecciRNAs and their roles in the mitochondrial entry of proteins. Sci. China Life Sci..

[18] Mercer T.R., Neph S., Dinger M.E., Crawford J., Smith M.A., Shearwood A.M. (2011). The human mitochondrial transcriptome. Cell.

[19] Nourbakhsh S.T., Mirzaei S.A., Mohamadhashem F., Naghizadeh M.M., Razavi A.N., Mansoori Y. (2025). Pathological expression of mitochondrial genome-derived circRNA SCAR/mc-COX2 and its ceRNA network in colorectal cancer: implications for clinical significance. BMC Cancer.

[20] Wang K.C., Chang H.Y. (2011). Molecular mechanisms of long noncoding RNAs. Mol. Cell.

[21] Liang H., Liu J., Su S., Zhao Q. (2021). Mitochondrial noncoding RNAs: new wine in an old bottle. RNA Biol..

[22] Kumarswamy R., Bauters C., Volkmann I., Maury F., Fetisch J., Holzmann A. (2014). Circulating long noncoding RNA, LIPCAR, predicts survival in patients with heart failure. Circ. Res..

[23] Santer L., Lopez B., Ravassa S., Baer C., Riedel I., Chatterjee S. (2019). Circulating long noncoding RNA LIPCAR predicts heart failure outcomes in patients without chronic kidney disease. Hypertension.

[24] Bongolo C.C., Thokerunga E., Fidele N.B., Souraka T.D.M., Kisembo P., Rugera S.P. (2022). Upregulation of the long non-coding RNA, LIPCAR promotes proliferation, migration, and metastasis of hepatocellular carcinoma. Cancer Biomarkers.

[25] Turkieh A., Beseme O., Saura O., Charrier H., Michel J.B., Amouyel P. (2024). LIPCAR levels in plasma-derived extracellular vesicles is associated with left ventricle remodeling post-myocardial infarction. J. Transl. Med..

[26] Dowran R., Sarvari J., Moattari A., Fattahi M.R., Ramezani A., Hosseini S.Y. (2017). Analysis of TLR7, SOCS1 and ISG15 immune genes expression in the peripheral blood of responder and non-responder patients with chronic Hepatitis C. Gastroenterol. Hepatol. Bed. Bench..

[27] Hon K.W., Zainal Abidin S.A., Othman I., Naidu R. (2021). The crosstalk between signaling pathways and cancer metabolism in colorectal cancer. Front. Pharmacol..

[28] Pavlova N.N., Thompson C.B. (2016). The emerging hallmarks of cancer metabolism. Cell Metab..

[29] Vyas S., Zaganjor E., Haigis M.C. (2016). Mitochondria and cancer. Cell.

[30] Grasso D., Zampieri L.X., Capeloa T., Van de Velde J.A., Sonveaux P. (2020). Mitochondria in cancer. Cell Stress.

[31] Gururaja Rao S. (2017). Mitochondrial changes in cancer. Handb. Exp. Pharmacol..

[32] Abdelmaksoud N.M., Abulsoud A.I., Abdelghany T.M., Elshaer S.S., Rizk S.M., Senousy M.A. (2023). Mitochondrial remodeling in colorectal cancer initiation, progression, metastasis, and therapy: a review. Pathol. Res. Pract..

[33] Wang S.F., Tseng L.M., Lee H.C. (2023). Role of mitochondrial alterations in human cancer progression and cancer immunity. J. Biomed. Sci..

[34] Liu Y., Sun Y., Guo Y., Shi X., Chen X., Feng W. (2023). An overview: the diversified role of Mitochondria in cancer metabolism. Int. J. Biol. Sci..

[35] Ren B., Guan M.X., Zhou T., Cai X., Shan G. (2023). Emerging functions of mitochondria-encoded noncoding RNAs. Trends Genet..

[36] Villegas J., Burzio V., Villota C., Landerer E., Martinez R., Santander M. (2007). Expression of a novel non-coding mitochondrial RNA in human proliferating cells. Nucleic Acids Res..

[37] Gao Y., Chen Z.Y., Wang Y., Liu Y., Ma J.X., Li Y.K. (2017). Long non-coding RNA ASncmtRNA-2 is upregulated in diabetic kidneys and high glucose-treated mesangial cells. Exp. Ther. Med..

[38] Zhang Z., Gao W., Long Q.Q., Zhang J., Li Y.F., Liu D.C. (2017). Increased plasma levels of lncRNA H19 and LIPCAR are associated with increased risk of coronary artery disease in a Chinese population. Sci. Rep..

[39] Wang H., Song T., Zhao Y., Zhao J., Wang X., Fu X. (2020). Long non-coding RNA LICPAR regulates atrial fibrosis via TGF-beta/Smad pathway in atrial fibrillation. Tissue Cell.

[40] Wang X., Li D., Chen H., Wei X., Xu X. (2019). Expression of long noncoding RNA LIPCAR promotes cell proliferation, cell migration, and change in phenotype of vascular smooth muscle cells. Med. Sci. Monit..

